# Metal-based nanoparticles, sensors, and their multifaceted application in food packaging

**DOI:** 10.1186/s12951-021-00996-0

**Published:** 2021-08-26

**Authors:** Antul Kumar, Anuj Choudhary, Harmanjot Kaur, Sahil Mehta, Azamal Husen

**Affiliations:** 1 Department of Botany, Punjab Agricultural University, Ludhiana, 141004 India; 2 International Centre for Genetic Engineering and Biotechnology, Aruna Asaf Ali Marg, New Delhi, 110067 India; 3 Wolaita Sodo University, P.O. Box: 138, Wolaita, Ethiopia

**Keywords:** Nanotechnology, Metal-based nanoparticles, Food processing, Antimicrobial properties, Low-density

## Abstract

Due to the global rise of the human population, one of the top-most challenges for poor and developing nations is to use the food produces safely and sustainably. In this regard, the storage of surplus food (and derived products) without loss of freshness, nutrient stability, shelf life, and their parallel efficient utilization will surely boost the food production sector. One of the best technologies that have emerged within the last twenty years with applications in the packaging of food and industrial materials is the use of green mode-based synthesized nanoparticles (NPs). These NPs are stable, advantageous as well as eco-friendly. Over the several years, numerous publications have confirmed that these NPs exert antibacterial, antioxidant, and antifungal activity against a plethora of pathogens. The storage in metal-based NPs (M-NPs) does not hamper the food properties and packaging efficiency. Additionally, these M-NPs help in the improvement of properties including freshness indicators, mechanical properties, antibacterial and water vapor permeability during food packaging. As a result, the nano-technological application facilitates a simple, alternate, interactive as well as reliable technology. It even provides positive feedback to food industries and packaging markets. Taken together, the current review paper is an attempt to highlight the M-NPs for prominent applications of antimicrobial properties, nanosensors, and food packaging of food items. Additionally, some comparative reports associated with M-NPs mechanism of action, risks, toxicity, and overall future perspectives have also been made.

**Figure:**
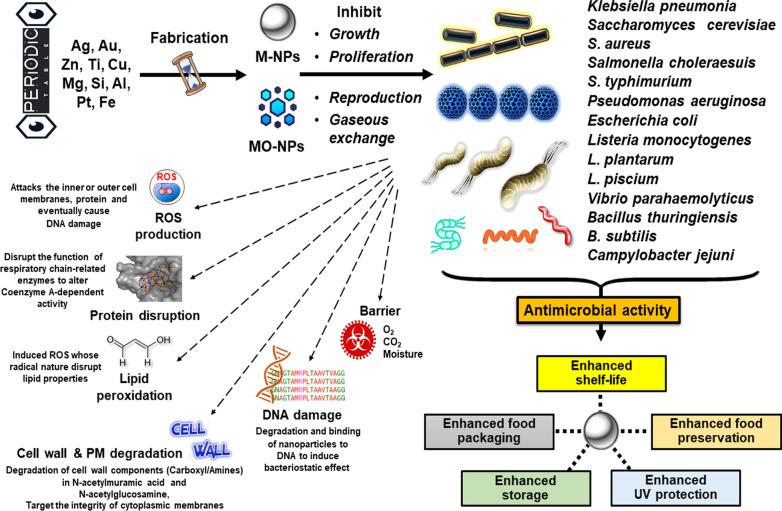

## Introduction

In the present century, food safety threats are emerging rapidly at an unpredictable rate [1]. Astonishingly, more than one-third of the total globally produced food gets wasted. This annual global food-as-waste value impacts the human’s stomach directly as it weighs around 1.3 billion tonnes. Interestingly, this food wastage also impacts the global economy as food wastage is worth nearly $1 trillion. This whopping number is so much big that this amount worth is equal to the upcoming USA infrastructure bill meant for future construction projects throughout the entire USA. According to the FAO’s Food Loss Index (FLI), approximately 14% of the total food produced gets wasted in the post-harvest stage only. Furthermore, according to UNEP Food Waste Index (FWI) report 2021, more than 900 million tonnes of food waste were produced in 2016 with a maximum share of 61% from households solely [2, 3]. Considering all these facts, the point is that substantial food stocks are not eaten by humans. This even puts enormous negative pressure on the environment, social status, and economics. Additionally, another frightening issue is the continued consumption of contaminated food products with very low nutrients value. This consumption eventually leads to various disease epidemics and threatens the food security of every nation. As a result, food-borne pathogens or spoilage needs intensive screening and monitoring to access the challenges in the food industries and or food safety [4–10]. Considering this, the issue of strengthening the food regulation system throughout the world was emphasized by the World Health Organization (WHO) in their slogan "farm to plate" put forward in 2015 [11].

Nanobiotechnology, the emerging field of biology has gained much attention since the twentieth century and has been established as an area with multiple applications [12, 13]. As per history, nanotechnology has brought the revolution after the efforts of a noble laureate, Richard P. Feynman, who also gave a lecture entitled "There's plenty of room at the bottom" in the year 1959. Since then, nanotechnology has been observed to take bigger and bigger leaps that have resulted in designing as well as manufacturing of characterized particle(s) in a significantly desired shape, size, and form [4, 5, 13, 14]. This can be evidentially supported by the fact that precision placement, controlled manipulation, measurement, modelling, and production of nanoparticles (NPs) in nanoscale is an easy task that has been pursued excellently on a global scale [15–18].

NPs are broadly classified into various classes based on their physical and chemical properties: Ceramics NPs, Carbon-based NPs, Semiconductor NPs, Metal NPs, Lipid-based NPs, and Polymeric NPs [18, 19]. The shape, size, facet, advanced optical properties (broad absorption band range), and excellent edge cutting (for example, coating of gold NPs) are primarily used in multiple avenues. One of the very prominent examples is the use of gold-coated NPs in the Scanning Electron Microscopic (SEM) technique for increasing the visualization efficiency for obtaining higher quality images [18–20]. Similarly, NPs have been utilized in the form of nanospheres, nanobelts, nanoplates, nanorods, nanoprisms, nanotetrapods, and nanocubes. These can be categorized into round decahedrons, face-centered cubic, star decahedron, regular decahedron, marks decahedron, cuboctahedron, and icosahedrons [21–23]. Interestingly, the use of NPs has also accelerated in applied and commercial microbiology areas due to various properties. These properties include high electrical conductivity, active surface, optical properties, specific ion release, resistance to crushing (near identical strength), stable agglomeration state, discrete energy levels, and durable stability under circulatory systems [4, 5, 8, 24–26].

As per the estimations by Institute for Health and Consumer Protection (IHPC), the NP-based market was predicted to touch the mark of $20 billion by the year 2020 [27]. These M-NPs show much advancement over synthetic chemicals used for food packaging and their preservation over a long duration of time. The unique characteristics, different physical and chemical properties, and microbial response to each NPs result in variation in practical approach in food packaging. Further, various types of M-NPs and their specific derivatives have gained enormous attention for their promising antimicrobial properties and empowered food industries in the past two decades [26, 27]. For example, multiple M-NPs such as zinc oxide (ZnO), magnesium oxide (MgO) copper oxide (CuO), silicon (Si), titanium dioxide (TiO_2_), calcium oxide (CaO), silver oxide (Ag_2_O), and gold (Au) have been already investigated for exhibiting the antimicrobial activity, and they also minimize biofouling [8, 19, 26–28].

Specifically, in terms of microbiological food perspective, nanotechnology has been effective in increasing food safety because of its significant role in every stage of the food chain ranging from food packaging, processing, preservation, quality monitoring to storage [8, 13]. Moreover, the use of NPs in nano-packaging components has been shown to improve long-distance packaging, storage product stability, and prevent deterioration from food spoilage [14, 15, 28]. Nano-components also have shown antimicrobial properties and restricted the growth activities of both pathogenic and spoilage microbes [12, 13, 22, 29] and hence, act as an emerging tool in food processing and preservation industries [29–31]. Due to nanomaterial and nanosensor-based assays, the easy identification of foodborne bacterial species including *Listeria monocytogenes, Escherichia coli,* and *Salmonella* spp. have also been reported [9–11].

NPs-food packaging materials are commonly made from polyethylene terephthalate (PET), polypropylene (PP), and non-degradable polyethylene (PE), where molecules penetration of H_2_O and O_2_ and is prevented. Additional, coatings containing flavors, antioxidants, antimicrobial agents, emitting sachets, and preservatives with the aim to safety and maintain food quality. It also offers antimicrobial active packaging, upgrading food stability, color, detection of food spoilage organism, the possibility of oxidation prevention, UV protection activity, and bio-based packaging [34]. For example, Silver-NPs (AgNPs) have increased antibacterial properties, Silica-NPs (SiNPs) in the plastic restrict the gaseous exchange that enhances the product’s shelf life, Nano-sized iron particles have increased reactivity and bioavailability. The incorporation of MNPs has significantly enhanced the shelf life of canned or preserved food with efficient management of spoilage. This has resolved the crises of food spoilage and ensuring food reaches to masses [35].

Furthermore, NPs have been used to improve the food quality and act as antibacterial agents including fullerenes, carbon nanotubes (CNTs), nano-ZnO, nano-Ce_2_O_4_, nano-TiO_2_, and nano-Ag that display antimicrobial characteristics [4]. In this regard, the extensive usage of nanosilver (nano-Ag) like NPs is evaluated as highly efficient control over various drug infectious or resistant microbes and confirms to be good in preventing organism evolving antibiotic resistance [5, 6, 10, 15]. For example, nano-Ag that was synthesized by a common “green” synthesis technique using leaf extract of *Plectranthus amboinicus* has been effective activity against the *Penicillium* spp. and *E. coli* [6, 7, 31]. Thus, based on the current information, the present review unfolds the sustainable green synthesis of NPs for prominent applications of antimicrobial properties, nanosensors, and food packaging of food items. An attempt has also been made to understand the M-NPs mechanism of action, risks, toxicity, and future perspectives.

## Green synthesis of NPs-An emerging and more sustainable approach toward mitigating the food packaging challenges

Several physical and chemical methods have been developed for the NPs fabrication and or synthesis. However, the deliberate synthesis of NPs by chemical/physical methods requires a fairly large amount of toxic chemicals which also leave undesirable materials that pollute the environment [28]. Therefore, in recent years, biogenic or green synthesis of NPS using fungi, bacteria, actinomycetes, algae, and higher plants have emerged as potential nanofactories which are also cost-effective and eco-friendly [36–38]. Additionally, it has been noticed that the NPs prepared using plant extracts are more stable, cheap, mono-dispersed, and takes less time to reduce [27, 28, 39]. Thus far, several reviews have been published on the fabrication and characterization of NPs [36, 38, 40–43].

It has been observed that several factors such as the concentration, pH, incubation time, and temperature of plant extract or biomass affect the conversion of metal ions to NPs and control the shape and size of NPs during fabrication. In a study, NPs biosynthesized from leaf extract of *Carica papaya* where CuO was used as a catalyst. It was showing the degradation of blue dye R-250 (Coomassie brilliant) under exposure to sunlight. The reduction in absorption intensity of dye enriched with CuO-NPs has been achieved without any fluctuation in actual absorption at 559 nm in the visible spectrum region after 2 h. Such degradation of dye has been dependent on the morphology and CuO-NPs size [27].

In another finding, CuO-NPs were derived from aqueous leaf extract of *Diospyros montana* and CuSO_4_. The plant-based extract has advantages over conventional or chemical methods. It does not need sterile conditions and cell culture maintenance. It also acts as a natural capping agent, helps in the stabilization of NPs along with the reduction of NPs-synthesis to a single step. The stabilization and reduction of such large-scale NPs production are achieved by the combined activity of various biomolecules such as flavonoids, terpenoids, phenolic acid, alkaloids, tannins, polysaccharides, enzymes, amino acids, and proteins [26, 40–42].

The derived CuO-NPs from leaf extract of *D. montana* was exhibited antibacterial activities against *P. aeruginosa, P. vulgaris, K. pneumoniae, E. coli, C. xerosis, S. epidermidis, S. viridans, S. pyogenes, S. mutans,* and *S. aurens.* The green synthesis of NPs has shown effective and efficient photocatalytic degradation of various dyes. It can be operated as a catalyst in the reduction of toxic materials, dyes, and industrial waste. Except for these, such NPs fabrication is inexpensive and easily available without selecting any organic solvent [26].

In another published article, high concentration anisotropic suspensions of AgNPs by soluble protein was hydrolysate or peptone using low volume but high amount nano synthesis and thus, increases the economic importance of green route-based synthesis. Numerous reports have been evidenced of the presence of multidrug-resistant bacterial types due to mutation, changing environmental conditions, and pollution. To overcome these issues, rapid upsurge in green route NPs is extremely needed, and such fabrications help to combats the microbial strain infections. [45].

Moreover, it offers the synthesis of specific microbial nutrients like peptone-coated AgNPs that proved to be economically and ecologically feasible nano-synthesis. The facile and rapid synthesis of peptone-coated AgNPs with desired parameters might give various advanced methods in the characterization of antimicrobial application. Bastos-Arreta et al. [40] used the grape stalk waste extract and suggested that they possessed components that can act as a stabilizer and reducing agent in NPs production. The AgNPs synthesized from leaf extract of *Eriobotrya japonica* were used in the catalytic degradation of reactive dyes. Several other plants and their extract has been used for AgNPs fabrication, such as *Areca catechu* [44], *Erigeron bonariensis* [45], *Momordica charantia* [46], *Euphorbia amygdaloides* [47], *Impatiens balsamina* [48], *Aloe vera* [49], *Artemisia absinthium* [50], *Chelidonium majus* [51], *Terminalia chebula* [52], *Cerasus serrulata* [53], *Solanum indicum* [54], *Fraxinus excelsior* [55] and so on. Quite often, the fabricated NPs have shown antimicrobial response [25].

Green route synthesis offers the great intrinsic capacity to oxidize, reductions of dyes, stabilization, highly negative zeta potential of synthesized MNPs, and biodegradability. Green synthesis of the M-NPs provides promising advantages with the least disadvantages reported so far [56–59] Likewise, the formation of M-NPs achieved through leaf extracts contains multiple involvements of primary and secondary metabolites. Therefore, it is very tough to evaluate the function of single metabolites at par. Furthermore, the synthesis requires multiple steps and may pass through several complementary chemicals to know the exact role of single metabolites [60, 61], thus some extensive investigations are required.

## Derivatives, properties, and mode of action

In the past two decades, metal oxides (MOs) and mixed metal oxides (MMOs) are the most studied material and explored recently in the field of food packaging. MOs display defects or extent of vacancies that distinguish the nature of MOs from each other. It also affects the oxygen partial pressure above MOs and causes a continuous alteration in equilibrium composition and lattice parameters. The d-block MOs (CuO, MgO, ZnO, and TiO_2_) have antimicrobial activities due to their minute variations in the O-atom defects and stoichiometry [62]. MO-NPs integration on polymers (bio-based or petroleum-based) alleviates superiority in the properties of the nanocomposites [63]. Several structural-based NPs and nanoscale MOs are utilized to modify the mechanical properties, thermal stability, and barriers in food packing.

However, Ag and Au are the most reliable metal and are extensively used due to their lack of reactivity. On the other hand, the oxides of Au and Ag are not getting much importance because of instability in nature [64]. For instance, Au_2_O_3_ has the heat of formation is 119.3 kJ/mol and implying its instability [65]. Oxidative stress and free metal ion toxicity are the well-known proposed mechanisms for antimicrobial activities [66]. Both of them (Ag-NPs and Au-NPs) have high photothermal activity, ease of detection, polyvalent effects, and high functionalization activity. It leads to their non-toxicity and antibacterial activity. These NPs bind to the bacterial membrane and induce the alteration of membrane potential. Hence, they reduce the ATP level and inhibit tRNA binding to the ribosome [67]. Au-NPs show a greater antibacterial response for the gram-positive bacteria than the gram-negative bacteria because of facile internalization in gram-negative bacteria [68].The Au-NPs biocidal properties are due to the dispersion extent in the medium and roughness. The potential functionalization of Au-NPs tagged them as “ideal NPs” for antimicrobial activities.

In contrast to Au-NPs, the Ag-NPs incorporation into the polymer matrix alters the antibacterial activity and gas permeability. The derivatives of Ag-NPs such as silver-embedded cellulose nanofibrils have a 10.72 ± 4.96 nm average size and peak for surface plasmon resonance absorption at 397 nm [69]. The Ag ions can also be released from its composite films and showing antimicrobial activity against *Listeria monocytogenes* and *E. coli*.

Most importantly, the Cu-NPs and CuO-NPs are the two main effective agents used in food packaging materials. CuO-NPs are more prominently exploited for reducing the fungal, bacterial, and viral growth in food packing [70]. The higher surface area of CuO-NPs has increased interaction with the cellular membrane. The CuO-NPs have shown antibacterial activity against both gram-positive and gram-negative bacteria in all bio-packaging systems. The bio-packaging systems involve copper incorporated chitosan, cellulose, agar, and poly (3-hydroxybutyrate- co-3-hydroxyvalerate) [71–74]. However, some polymeric systems (CuO and chitosan nanofibers) have been observed to show synergistic effects [72]. It is well known that the size, surface area, structure, morphology, and variation in oxidation states confer antimicrobial activity of MOs including CuO. The coupling or doping of CuO with other metals or MOs can also potentiate the overall properties of packaging material.

Interestingly, the TiO_2_-NPs are highly thermo-stable materials with modified properties in biodegradable films. TiO_2_-NPs in the food grades are photostable, cheap, non-toxic, and have antimicrobial activity. The controlled size and colloidal suspension can be attained with help of wet chemistry methods [75]. The polymer incorporated TiO_2_-NPs have several properties such as photocatalytic activity, mechanical, physical, and thermal properties along with anti-bacterial properties. The used polymers are chitosan, polylactic acid, high-density polyethylene, and starch. The photocatalytic activities are based on the adsorption of the N(III) atom of the imidazole ring on the Ti(V) atom, therefore, high and stable energy adsorption takes place. Thus, TiO_2_ induces the photocatalytic degradation of ornidazole and confers the catalytic degradation at a microscopic level [76].

Furthermore, ZnO is another novel material at the nano-sized level which is considered as a safe antimicrobial material for next generations food preservatives. US Food and Drug Administration has been listed ZnOs as a food additive and approved as a generally recognized as safe material [77]. ZnO-NPs are potent materials because of their antimicrobial and photocatalytic activities. The photocatalytic activity is based on morphology and can be altered from spherical one to hexagonal nanodisks or nanorods [78]. ZnO-NPs have improved properties concerning crystalline structure, chemical composition, morphology, surface functional chemical group, and specific surface area.

Apart from the above-mentioned MOs, MgO is another oxide that has high thermal conductivity and low electrical conductivity. It even has high stability that confers strong antimicrobial properties. Apart from this, it displays impermeability to gas, flexibility, thermal stability, recyclability, and hence, MgO-NPs based packaging material has substituted the several construction materials of food packaging [76].

Similarly, the Mg-NPs incorporated in biodegradable polymers has improved properties (such as mechanical, optical, barrier, and thermal) besides antimicrobial activity. For instance, the polylactic acid-MgO-NP nanocomposite films have improved 22% tensile strength and 46% plasticity using 2% MgO. Additionally, the water vapor and oxygen barrier properties are also improved by 57% and 65%, respectively [79, 80].

Numerous NPs like TiO_2_ and ZnO are frequently utilized as photocatalyst agents to degrade microorganisms and organic molecules whereas layered silicates, nano-clays, and Ag-NPs can be toxic to food spoilage pathogens [81, 82]. The photocatalytic activities of nano-TiO_2_ and nano-ZnO are contributed to ROS generation and lead to cytoplasmic oxidation of microbial cells terminated with cell death. Ag-NPs can attach to the cell surface and deteriorate the lipo-polysaccharides, thus, forming a membranous pit on the cell wall [82]. It has also been suggested that Ag-NPs produce ROS and free radicals which cause apoptosis leading to cell death, thus checking their replication [25]. The Ag-NPs plastic polymer complex such as zeolite having low density compounds can enhance shelf life and inhibits microbial growth in orange juice. However, such an active nanocomposite complex has been proved as a good antimicrobial agent with high temperatures. ZnO-NPs show diverse inhibition against *Salmonella aureus, E. coli* and *Bacillus atrophaeus* and are even much attractive for packaging. Under high UV conditions, ZnO can liberate a higher amount of hydrogen peroxide that causes oxidative stress in microbes [82].

To evaluate the decay rate, various indices are marked such as pyrogallol peroxidase (POD), ethylene, malondialdehyde (MDA), and polyphenol oxidase (PPO). Under UV radiation, ZnO can oxidize ethylene into carbon dioxide and water and decreases the accumulation of POD, ethylene, MDA, and PPO that enhance the shelf life of food products [83]. Conclusively, ZnO is comparatively more attractive and efficient than Ag-NPs due to its cost-effectiveness and low toxicity. European Food Safety Authority approved food contact like nano-TiN is derived via the heating of TiO_2_ in nitrogen gas at high temperature and is chiefly used as a packaging agent [83, 85].

## Efficacy of metal based-nanoparticles (M-NPs) as sensors

**Fig. 1:**
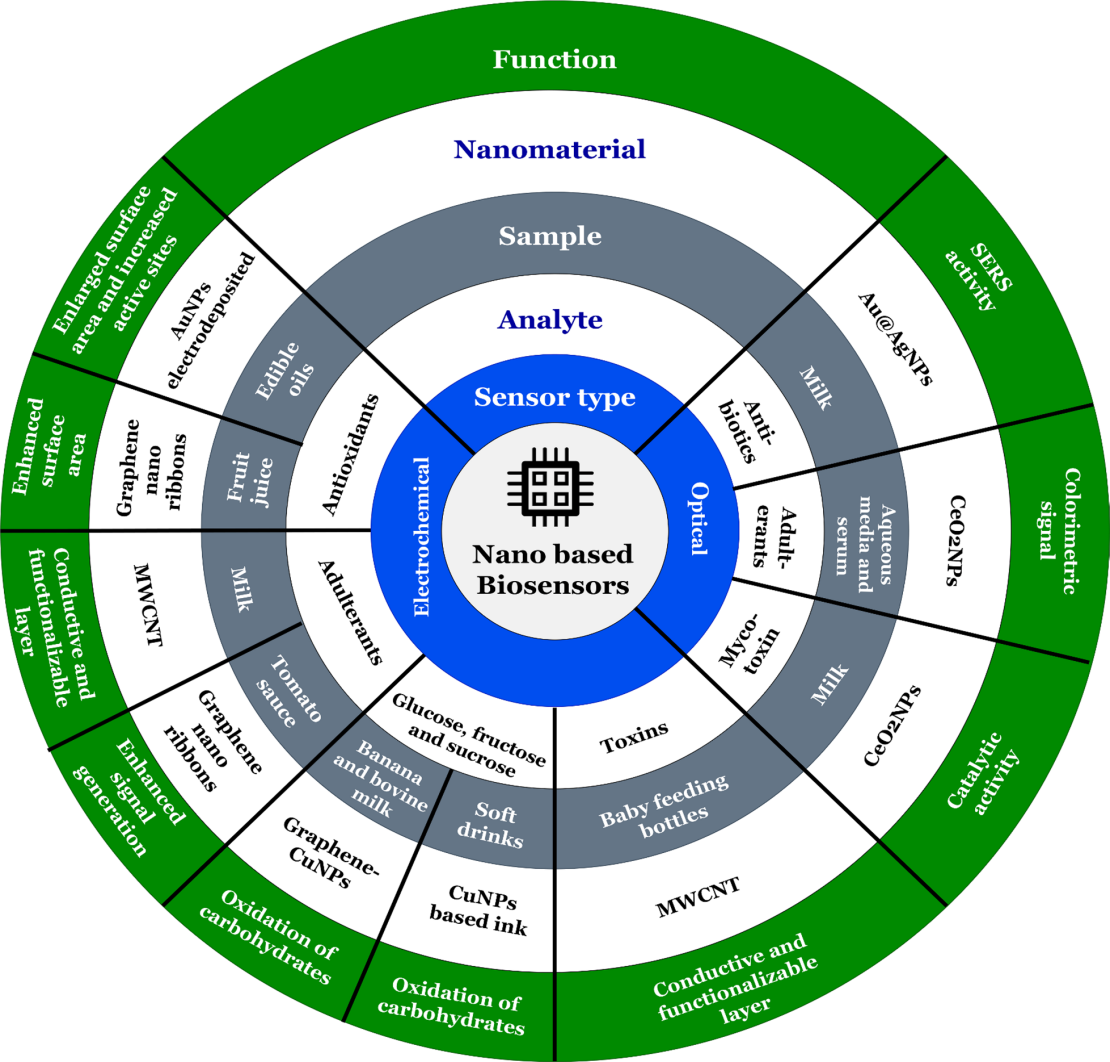
Overview on different types of nanosensors and their functional role in the preservation of food products

In the food packing industry, nanotechnology provides promising solutions for increasing the shelf-life period, safety from microbes, and quality assurance [33]. The M-NPs have metal as a precursor molecule that is produced by either chemical, physical or biological methods. While the fabrication of M-NPs, various nano-based inorganic materials are employed including gold (Au), silver (Ag), zinc (Zn), titanium oxide (TiO_2_), silicon oxide (SiO_2_), magnesium oxide (MgO), and zinc oxide (ZnO) (Fig. 1). They can either make direct contact or migrate slowly to react with food organic materials [29, 30].

Metals such as Ag and Au are considered excellent materials because they have high plasmon resonance [31, 31, 32, 86]. The M-NPs have a higher surface area and thus brought higher activity to the surface plasmons. Therefore, this property of the plasmon resonance phenomenon is being used for employing it as a sensor for identifying the diverse molecules present in the environment [87]. Moreover, advancement in nanotechnology helps in the transport of nano-metallic compounds effectively in food products [87] and ensures their quality via inhibiting the growth of food spoilage agents (Table 1).

**Table 1:** Recent studies (during 2018–21) on M-NPs based technologies for preservation and packaging of food materials

Metal/Metal oxide used	Carrier compounds	Edible product	Concentration used /Properties of M-NPs	Technique used	Results	Drawbacks	References
Ag ZnO	Low-density polythene Low-density polythene	Orange juice Orange juice	1.5% of Ag 1% of ZnO	Thermal processing method Thermal processing method	Decreased mold and yeast growth, improve the quality of juice Decreased concentration of *L. plantarum*, enhance juice quality	Silver nanoparticles damage DNA damage Zn nanoparticles decrease the ascorbic acid content	[160]
Ag ZnO and Ag Pullulan and Ag	Absorbent pad Cellulose pad Low-density polythene Low-density polythene	Meat Meat Meat Turkey Meat	0.1 and 1% 1% – 40-100 nm	Engineered Fibres Technology Physical (thermal/UV) methods	Effective reduction in the growth of *E. coli* and *S. aureus* Considerable decreased the microbial growth, improves the product quality Inhibitory effect on bacterial growth Suppress growth of *L. monocytogenes* and *S. aureus*	– Less evident on the yeast cells during bacterial growth – Less evident on the yeast cells during bacterial growth	[161]
Ag, Kaolin, TiO_2_ Ag	Polyethylene Polyvinylallyl nanofibrils	Chinese-jujube Lemon	30% Nanopowder (35% Ag, 25% Kaolin, 40% TiO_2_) 5%	– –	Increased fruit shelf life for a longer period and also maintaining its quality Compared with typical conventional coatings, the nanofiber film may have potential applicability and high antibacterial capability against *E. coli* and *S. aureus*	– –	[162]
Ag Cu ZnO Ag and essential oil Ag, Cuo, and ZnO TiO_2_, Ag	Cellulose pad Cellulose absorber Polyvinyl chloride Pullulan films Low-density polythene Polylactic acid matrix	Melon Juice of melon and pineapple Fuji apple slices Meat Cheese Cheese	1% 1% 0.1% 2% essential oil, 100 nm ag nanoparticles Ag (35 nm), CuO (50 nm) and ZnO (30-50 nm) 10 nm	Thermal processing method Physical (thermal/UV) methods Disc diffusion method –	Decline in the growth of microbial activity, maintain fruit freshness for longer intervals Reduced fungal activity, Inhibit the growth of molds and yeast Improve quality by inhibiting the growth of *E. coli* Antibacterial activity (*S. aureus* than *L. monocytogenes)* Considerable decrease in coliforms Inhibits total bacterial count	Less evident on the yeast cells than during bacterial growth Impacts of AIT in combination with other compounds on the organoleptic properties of LEW – Less evident on the yeast cells than during bacterial growth Decrease effect quality of product	[163]
ZnO	Nisin, Allyl isothiocyanate Poly-lactic acid	Egg albumin –	250 mg 3%	Thermal processing method Silanization	Inhibit the growth of *Salmonella sp.* Reduce microbial activity	– –	[164]
Ag films Ag	Hyperbranched polyamide-amine Polyvinyl-pyrrolidone	Cherry tomato Asparagus	2.0 mM 1.5/100 ml	– –	Strong antibacterial effect on *E. coli* and *S. aureus* Maintain quality of fruit, and inhibit the growth of psychrotrophic aerobes	Effect the firmness of fruit –	[165]
ZnO	Chitosan	Papaya	0.1%	–	Regulates the activity of *S. aurens* and *E. coli* Improve fresh-cut postharvest quality	–	[166]
Ag Ag_2_O Ag	Absorber Polyethelene Low-density polythene Furcellaran	Juice of kiwi and melon Apple juices Apple pieces Kiwi	1% 7% 2% 0.1 mM	Physical methods (UV, heat) and chemical methods (sodium borohydride) – – Casting method	Inhibit the growth of molds and yeasts Suppress bacterial growth against *A. acidoterrestris* Restrict browning of apple slices and prevent microbial growth Bacteriocidal activity against *S. aureus* and *E. coli*	Inhibitory action of Ag on average proteins content Nanoparticles decrease the ascorbic acid content Weight loss occurred –	[167]
Ag, Kaolin, TiO_2_	Low-density polythene	Strawberry fruit	30% Nanopowder (35% Ag, 25% Kaolin, 40% TiO_2_)	–	Fruit preservation for a longer time ensuring its quality at the post-harvest stage	Decline in the contents of total soluble solids, titratable acidity, and ascorbic acid	[168]
Nanoclay	Carboxymethyl cellulose polyvinyl alcohol film	Walnut	3%	–	Prevent the fruit for longer time preservation	Decrease fruit quality	[169]
Ag ZnO	Chitosan Bacterial cellulose	Poultry Meat Poultry meat	6% 150 nm	– Chemical polymerization	Prevent bacterial growth and enhance shelf life Prevent microbial activities	Nano-layers are fibrous and can easily get damaged due to mishandling –	[170]
Ag and polyvinyl chloride	Low-density polythene	Bread	5%	Polyethylene terephthalate	Antibacterial properties against two pathogens *L. monocytogenes* and *E. coli*	Organoleptic properties of the food	[171]
Ag	Gelatin	Grapes	0.1%	Nanocomposite film	Enhance shelf life of red grapes up to 14 days, gas and moisture barrier properties	–	[40]
Ag + montmorillonite film	Nanocomposite clay blend film	Chicken sausages	40 mL	Solvent casting method	PAGM film exhibited potent antibacterial activity against *S. typhimurium* and *S. aureus*	Inhibition of bacterial growth and control polythene pouch failed	[172]
Ag	PVA-montmorillonite K10clay nanocomposite blend film	Chicken sausages	40 mL of 1 M AgNPs	Photo-assisted method	Extending the shelf life of chicken meat Reducing the microbial activity of *S aurens* and *S. typhimurium*	–	[173]
Ag and Ecoflex films	Composite films	Meat	1.5%	3D printing process	Lower concentration of *S. enteritidis*	Decline quality traits	[174]
Ag, TiO_2,_ and clay NPs Ag	Low-density polythene Low-density polythene	Chicken Carrots	1% 5000 ppm	– Ion exchange reaction method	Maintain the quality and shelf life Shelf life prolongation of fresh-cut carrots by controlling both microbial and sensory quality during the refrigerated storage	– –	[175]
Ag	Low-density polythene	Carrot	2.5%	MAP technology	Antimicrobial properties	Decreased ascorbic acid content	[176]
Ag Ag–Cu NPS with essential oil	Polyethelene Linear low-density polyethylene	Mushrooms Chicken	0.1 M 4%	TAXT Express-v3.1 texture analyser Batch mixer Brabender	Decrease concentration of yeasts and molds, decrease microbial counts, such as pseudomonas, mesophilic, psychrophilic Maximum antimicrobial activity against *L. monocytogenes*, *S.* *typhimurium*, and *C. jejuni*	Effect both quality and quality Nanoparticles produce cellular toxicity, oral toxicity, inflammation, and skin toxicity	[177, 178]
Ag	Cellulosic sheets	Cabbage, tomatoes	10%	–	Use of AgNPs in preventing the growth of foodborne pathogens and elevate shelf life	–	[179]
Ag	Sodium alginate films	Carrot, pear	0.1 M	–	Inhibitory effect against *S. aureus* and *E. coli*	–	[180]
Agar, alginate along with Ag TiO_2_, Ag, essential oil	Ternary blend hydrogel films Polylactic acid matrix	Potatoes Mangoes	Silver nitrate (4.72 g in 100 mL) Agar, alginate, and collagen powder (3 g each) 3%	Solution plasma process –	Strong antimicrobial activity, prevent green coloration of potatoes during storage Reduce the bacterial growth and enhance quality	– Decrease ascorbic acid content	[181]
Ag	Polyvinylchloride	Beef	40-50 nm	–	Inhibit microbial and bacterial growth	–	[170]
Ag	Low-density polythene	Pork	3-20 nm	Polyethylene films	Suppress the growth of *B. thermosphacta L. sakei,* and *L. piscium*	–	[182]
TiO_2_ and Ag	PLA nanocomposites	Mangoes	–	–	Extending postharvest life up to 15 days	–	[183]

### Au-NPs

The Au-NPs are simply bioconjugated to DNA, aptamers, antibodies and therefore, used for bio-detection. Furthermore, the electromagnetic and unique optical properties allow them to enhance the sensitivity of the sensor [88]. For instance, melamine is an adulterant used in the milk to increase the nitrogen content and gives false readings for the total protein content in the milk. The gas chromatography-mass spectroscopy (GC–MS) is a standardized procedure for the detection of melamine recommended by the US Food and Drug Administration. However, in comparison, the Au-NPs are simple, highly sensitive, and cheap options for quantifying melamine as they have a quantification limit of 0.05 mg/L that exceeds the limit of 0.05–10 mg/kg for the GC–MS method (Table 1). The red color of Au-NPs colloidal solution is imparted under the absorbance of 523 nm that confirms the proper Au-NPs dispersion [89, 90]. The presence of melamine in the milk induces conjugation with Au-NPs and imparting blue color at a 640 nm absorbance value [90]. The chemical resonance energy transfer is considered to be responsible for high sensitivity for melamine detection.

Another example of milk adulteration is the addition of detergent (1.2 g/L in water). Methylene blue and azure A are the organic dyes that are used for detecting detergent supplementation in milk. Because of the thousand times higher value of extinction coefficient, Au-NPs are also employed for detecting the anionic component of detergents in milk [90]. Apart from non-biological adulteration, the NPs have been reported to be identifying various food pathogens. For example, milk is rich in nutrients and highly prone to pathogenic attacks. The Au-NPs combined with propidium monoazide-asymmetric polymerase chain reaction (PCR) is used to detect the emetic *Bacillus cereus* in the milk. Au-NPs conjugated with the long genomic DNA fragments (amplified by PCR) are stabilized by adding NaCl. After few minutes the visual detection is possible or with the help of a UV spectrophotometer [89, 91]. Similarly, *Salmonella* is detected in the milk with the help of isothermal recombinase polymerase amplification and unmodified Au-NPs combination (Table 1). This method is rapid, accurate, and cost-effective as compared to other standardized methods [92, 93].

Furthermore, Au-NPs have been applied for detecting meat spoilage. The tyramine, histamine, cadaverine, and phenylalanine are the biogenic amines produced by the bacterially mediated amino acid decarboxylation. The false smell is remained cryptic due to their preservation at 5 °C. In a report, Au-NPs were used to measure the histidine and histamine in the chicken meat at detection limits 0.6 μM and 6.59 × 10^–4^ [90]. Recently, the metal nanosensors were commercially exploited and gain much attention in food preservation summarized in Table 2.

**Table 2:** Recent advancements (during 2018–21) in metallic sensors tested to ensure the quality of food products

Edible product	Testing period (days)	Sensor used	References
Wheat Bread Red-Grapes	15	Polyvinylchloride, Ag-NPs	[40]
	25	Polyvinyl-chloride, Ag-NPs	
Turkey Meat	21	Pullulan films, Essential oils, Ag-NPs	[161]
	12	Coated films with Ag-NPs	
Kiwi	8	Furcellaran gelatine films, Ag-NPs	[167]
Beef	14	Ag-NPs, Noncellulose polyvinyl alkyl films	[163]
Tomato	9	Ag-NPs, polyamide-amine films	[172]
Pork	15	Polyethylene films along with liposomes-essential oil-Ag -NPs	[182]
Meat	4	Polyvinylallyl, Ag-NPs, montmorillonite films	[162]
Meat	28	Ecoflex, Silica carbon Ag-NPs	[174]
Litchi	7	Ag-NPs films	[184]
Chicken	5	Polyethylene with Ag, Clay, TiO_2_	[179]
Mushroom	21	Polyethylene with Ag-NPs	[177]
Carrot	10	Ag-NPs with low-density polyethylene	[176]
Chicken	21	Ag-NPs with low-density polyethylene	[178]
Lemon, Strawberry	10	Polyvinylallyl nanofibres, Ag-NPs	[162]
Tomato, Cabbage	7	Cellulosic sheet incorporated with Ag-NPs	[179]
Pear, Carrot	10	Ag-NPs along with Na-alginate films	[180]
Potato	10	Ag-NPs incorporated with agar or alginate	[181]
Poultry Meat	9	Ag-NPs along with polyvinyl allyl films	[185]
Saffron	180	Polyethylene films with Ag-NPs	[41]
Pistachio	8	Low density polyethylene with Ag-NPs	[186, 187]
Olive oil	–	Polyvinyl alcohol chitosan film with TiO_2_	[188]
Almonds, walnuts	365	Polyethylene Ag-NPs film	[169]

### Ag-NPs

In spite of the chemical stability, the Ag-NPs are a better choice than the Au-NPs. It is because of sharper extinction bands, higher extinction coefficients, higher scattering to extinction ratio, and also higher field enhancement. However, recent developments have been made to enhance chemical stability by focusing on optical properties [94]. The stable colloidal synthesis of Ag-NPs is mediated with the help of optimized AgNO_3_ and NaBH_3_ concentrations in a factorial design. The interaction between Ag-NPs and the amine group is strong and enhances the sensitivity of melamine detection. The detection of melamine is noted as yellow to red color change as surface plasmon bands fall at a longer wavelength. Since there are no stabilizing agents; hence, the detection limit is 0.009 mg/L [89].

The Ag-NPs can also be used in food spoilage detection. For example, in one of the published article, the cysteine and histidine incorporated Ag-NPs were applied to detect the lactic acid in the fresh milk with no color change. The imidazole group of histidine and thiol group of cysteine bonded Ag-NPs to lactic acid and resulted in NPs aggregation along with detected color change [95].

Ag-NPs based detection is very efficient in the assessment of post-harvest spoilage of agricultural and horticultural crops. For instance, the *Bacillus subtilis* causes spoilage in the *Musa acuminata* and produces 1,2-Benzenedicarboxylic acid, bis (2-methyl propyl) ester (a volatile compound) during its spoilage. The colloidal solution Ag-NPs is when applied to such spoiled bananas turned into reddish-brown color. Similarly, this method is also applicable to other fruit crops (Table 2) [96]. For instance, the spoilage of onion in the post-harvest period can be detected based on the release of organosulfur compounds. The application of Ag-NPs changes its color from yellow to orange initially and followed by an increase in spoilage it becomes colorless. The sensitivity and specificity of the sensor have been confirmed by the calorimetric analysis and UV visible spectroscopy for volatile sulfur compounds [97].

### ZnO-NPs

Devi et al. [98] had developed a chitosan-modified ZnO-NP-based sensor with xanthine content. The xanthine oxidase bounded on the carbon nanotube multilayer system associated with ZnO. The detection limit was observed to be 0.01 mM. Further, in the chicken thigh, ZnO is used to restrict microbial growth and regulates pH value [110]. However, Ag-ZnO (0.25% and 1%) helps to detect the growth of *Lactobacillus plantarum* in orange juice and causes significant *Salmonella* inactivation, mold counts, and decreases permeability using polylactic acid [99, 106]. The antimicrobial activity is elevated by exposure to visible light and it with decreasing NPs size [151].

### Other metal NPs

Apart from the Ag, Au and Zn-based NPs, the next most important NPs are of TiO_2_-NPs which have been also employed in detecting the pathogen contamination. TiO_2_-NPs immobilized *Salmonella* in the buffer solution and absorb UV light. The absorption is inversely proportional to the concentration of *Salmonella* bacteria which is showing high sensitivity and act upon a very low concentration of bacteria population [99]. A TiO_2_ and graphene composite has been developed to check the freshness of meat. It also evaluates the oxidation activity of xanthine oxidase [98, 100]. Except these, TiO_2_-NPs can be used to detect the pathogenic bacteria produced in the milk. The antibodies coupled with NPs are used to capture the *Salmonella* bacteria present in the milk. The external magnetic field has been applied to separate bacteria from analyzing samples.

Similarly, ZnO*–*SnO_2_ nanocomposite has been developed to check the shelf-life of fish and meat in preserved form. The sensor shows high sensitivity to the xanthine (purine decomposed product) [101, 102]. No doubt, Au-NPs, and Ag-NPs are highly exploited in the area of metal-based nanosensors. Besides these, several studies have been given to show the role of other sensors in food security. For example, a dendritic PtNPs-based sensor is developed to detect the bisphenol A (BPA) presence [103]. BPA is leached from the inner protective resin coatings of canned foods, polycarbonate tableware, water bottles, baby bottles, and food storage containers. Thus, it accesses the quality of food and migration of BPA from the packing.

## Functional M-NPs in food packaging

Several products are available on the markets to preserve food such as (i) nanoencapsulation of canola oil for fortified phytosterols (ii) Fortified fruit juice manufactured by High vive company for the encapsulation of fortified theanine, lycopene, and vitamin (iii) Life vitamins supplements used for nanoencapsulation of fortified vitamin beverages (iv) Daily boost used for encapsulation of bioactive components beverages (v) Nanoceuticals Slim Shake Chocolate, Apocarotenal, Paprika nanoemulsions or Beta-carotenal is used for the nanoencapsulation that help to enhance the flavor of the shake [104].

**Fig. 2:**
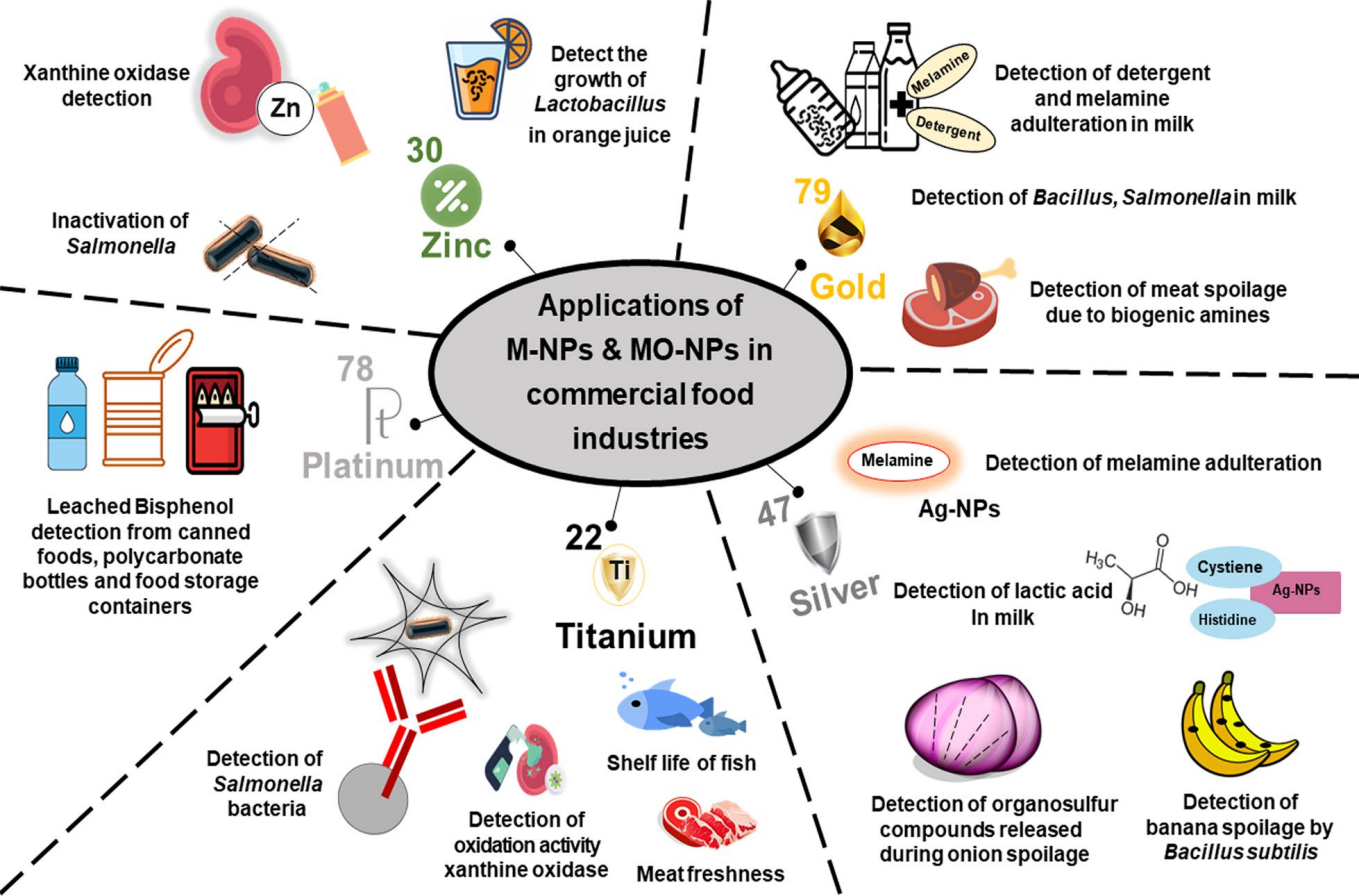
Metal nanoparticles and their feature used in commercial food industries

The employing of M-NPs in food packaging can suppress the activities of several microorganisms including *Alicyclobacillus acidoterrestris*, *Staphylococcus aureus, Escherichia coli, Lactobacillus plantarum,* and yeasts [105–108]. The M-NPs based nano-food packaging formulations include mass/heat transfer, nanobiotechnology, molecular synthesis, and nanoscale reaction engineering. These polymeric nanocomposites can be used in nano-food packaging and help them from packaging barriers like moisture, carbon dioxide, oxygen, ethanol, and improving emission of and flavors [105]. Several other advantages of M-NPs nano packaging are intelligent packaging, degradable biopolymers, and active packaging (Fig. 2) [44, 109–113].

### Role of metals and their forms in food packaging

#### Au-NPs

Au-NPs have shown their potential as antimicrobial agents and are used in the packaging of food products. Such packaging allows regulating microbial proliferation, oxygen control via enhancing the shelf life of packed material. The M-NPs along with PLA compound (polylactic acid biopolymer) and cinnamon oil increased mechanical strength, barriers, and the natural constitution of PLA.

The antimicrobial activities against *Listeria monocytogenes, Campylobacter jejuni,* and *Salmonella typhimurium* inoculated in meat samples were identified. The Ag-CuNPs films exhibited antimicrobial activities against the bacteria. In another study, the Ag-NPs were incorporated with hydroxypropylmethylcellulose/ tragacanth/ Beeswax against the activity of gram-negative (*Klebsiella pneumoniae* ATCC-10031, *S. typhimorum* ATCC-14028, *E. coli* ATCC-8739, and *Pseudomonas aeruginosa* ATCC-9027,) and gram-positive (*L. monocytogenes* ATCC-7644 *Streptococcus pneumoniae* ATCC-49615, *Bacillus cereus* ATCC-1247, and *S. aureus* ATCC-25923) and confirmed the dose-dependent inhibitory effect for both gram-negative and gram-positive bacteria [114]. However, prepared nanocomposites of Laponite/Ag-NPs/Carrageenan were encapsulated with the film of polypropylene-oxygen plasma surface also displayed the same behavior.

Moreover, Ag-NPs were prepared from the extract of *Digitalis purpurea* that assessed mechanical barrier, adhesion, and antimicrobial properties of nanocomposites. It remarkably enhanced the susceptibility of *S. aureus* and *E. coli* toward these nanocomposites [115]. Similarly, Kim et al. [116] reported that Ag-NPs were synthesized biologically derived from *Nigella sativa* extract prepared with chitosan-based nanocomposite films. Such nanocomposite films were developed pH-dependent persistent Ag-NPs release and assessed the efficacy of films against two gram negative bacteria (*P. aeruginosa* and *E. coli*) and two gram positive bacteria (*S. aureus* and *B. subtilis*). Thus, indicated notable antibacterial properties depend upon the concentration of Ag-NPs that retards bacterial growth (Table 3).

**Table 3:** Highlights illustrating the antimicrobial activities of synthesized M-NPs against microbial and fungal strains

NPs	Range (Size)	Source	Antibacterial activity	Antifungal activity	Shape(s)	References
CuO-chitosan	23.17 nm	*Sterculia foetida*	*E. coli*, *P. aeruginosa*, *K. pneumoniae*, *E. faecalis*, *S. flexneri*, *S. typhimurium*, *P. vulgaris*, *S. aureus, E. coli,* and *E. faecalis*	*A. terreus*	Crystal shaped	[205]
Cu-chitosan	2–350 nm	*Lawsonia inermis* and *Terminalia arjuna*	*S. paratyphi*, *B. subtilis*, *P. aeruginosa*, *S. choleraesuis*, *C. albicans, K. pneumoniae,* and *P. aeruginosa*	*F. oxysporum* and *P. capsici*	Cube-shaped	[206]
Ag-NPs	10-20 nm	*Medicago sativa, Cymbopogon flexuosus, Eucalyptus macrocarpa,* and *Jatropha curcas*	Methicillin-resistant *S. aureus* (MRSA), *M. smegmatis,* and *S. mutans*	*M. luteus*, *S. aureus, P. aeruginosa*, *E. coli*, *A. fumigates*, *A. niger*, *C. albicans* and *C. glabrata*	Spherical and rod-shaped	[207]
ZnO	50-60 nm	*Cassia fistula* and *Melia azadarach*	*E. coli, P. aeruginosa*, *S. aureus, B. subtilis,* and *S. mutans*	*B. cinerea and P. expansum*	Rectangular-shaped	[208]
MgO	36.7 nm	*Amaranthus tricolor, Amaranthus blitum,* and *Andrographis paniculata*	*S. dysenteriae*. *S. aureus,* and *E. coli*	*C. albicans*	Spherical, hexagonal-shaped	[209]
CaO Si	18.98 nm 100 nm	Chicken eggshell Sugarcane bagasse	*E. coli,* and *S. epidermidis* *S. aureus,* and *E. coli*	*P. aeruginosa,* and *C. tropicalis* *A. solani*	Fibre shaped Spherical shaped	[210]
TiO_2_	500 nm	*Moringa oleifera* and *Morinda citrifolia*	*S. typhi, E. coli,* and *K. pneumoniae*	*C. albicans*	Spherical shaped	[211]
Au-NPs AgO	20-100 nm 45 nm	*Rhodococcus* sp. *Plectonema boryanum* *P. boryanum, Thermomonospora* sp., *S. mutans*. *F. oxysporum,* and *Cyanobacteria sp.* *Eupatorium odoratum and Artocarpus heterophyllus*	*S. mutans* *R. mucilaginosa, E. coli, Pseudomonas, P. mirabilis, E. faecalis,* and *S. aureus*	*Candida sp.* *A. niger, A. fumigatus, and A. flavus*	Cubic, spherical, octahedral, hexagonal, pentagonal, truncated, and triangles Spherical and circular disc-shaped	[212]
Au-Pt	–	*–*	*E. coli*	*–*	Wire and chain shaped	[213]
Fe_2_O_3_	10-90 nm	*S. oneidensis* MR-1 and yeast cells	*S. aureus, B. subtilis*, *E. coli* and *P. aeruginosa*	*T. roseum, P. chrysogenum, A. niger, C. herbarum* and *A. alternate*	rhombohedral/ irregular and wormhole shaped	[214]
Cu	25 nm	*Gloriosa superba*	*S. aureus* and *E. coli*	*Saprolegnia* species	Cube and hemisphere shaped	[215]
Pt	4-13 nm	*Diopyros kaki*	*E. coli* and *A. hydrophila*	*C. acutatum, P. drechsleri, C. fulvum,* *D. bryoniae and P. capsici*	Spherical shaped	[216]

**Fig. 3:**
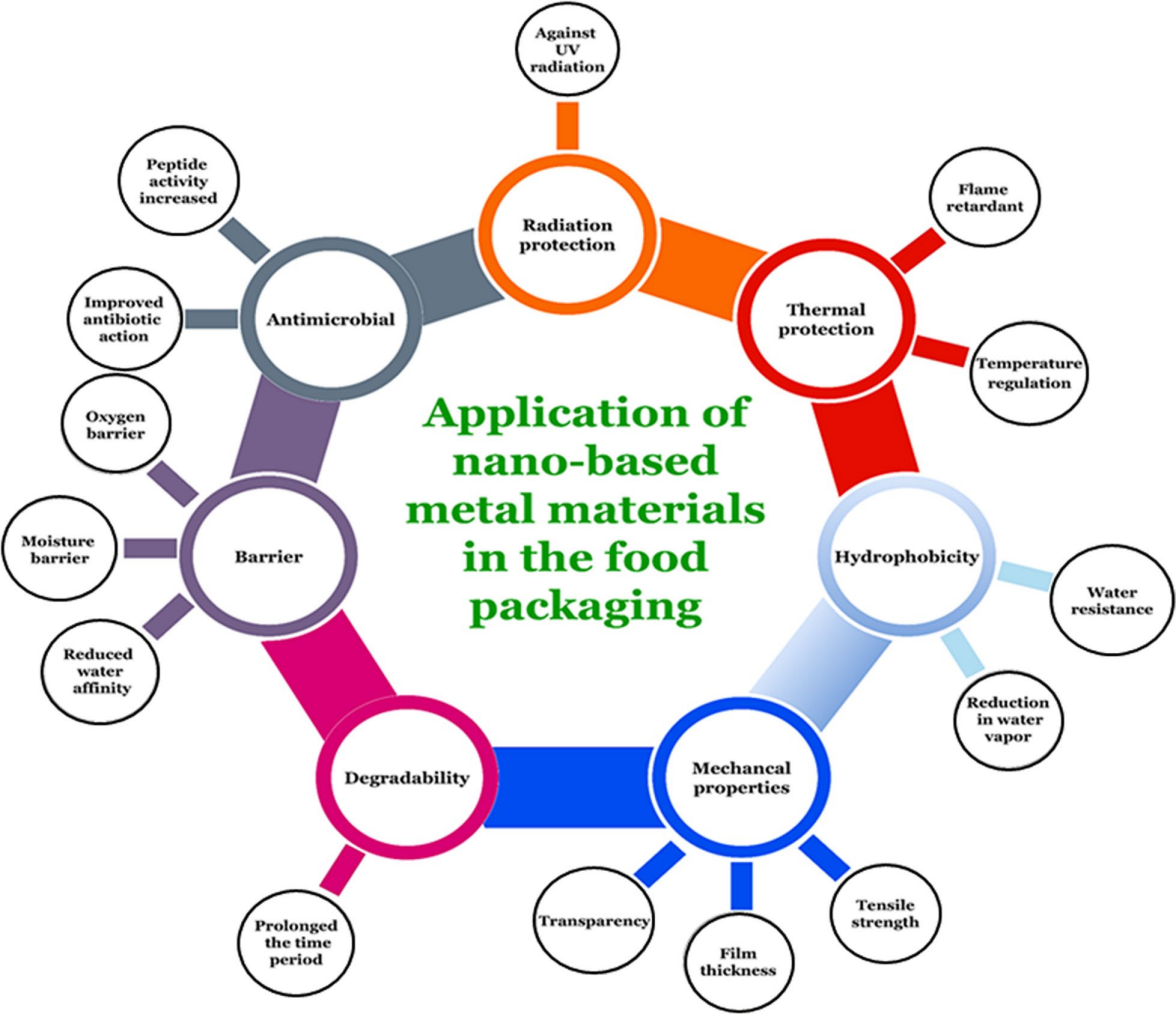
Applications and characteristics of nano-based metal-materials in food packaging

Recently, the Ag-NP layer of the chitosan-based composite prepared via 30%-aqueous chitosan solution along with 70%-polyvinyl alcohol and Ag-NPs was utilized during food packaging and showed antibacterial activity against food poisoning bacterial species [116]. Ag-NPs offer a fundamental mechanism related to antimicrobial properties by causing direct damage to membranes and cell walls of microbes. Such reactions with hydrogen ions and dissolved oxygen executed an oxidized surface and explored its significant role in the antibacterial properties (Fig. 3).

Additionally, nano-Ag has also displayed the property of a “two-layered barrier” toward water-borne pathogens in water treatment plants by showing antimicrobial activity and refining the water quality. As a result, commercial devices based on nano-Ag have been effectively utilized in ceramic microfilters against harmful pathogens in water purification systems [2, 6]. Recently, a more detailed study was conducted on deciphering the antimicrobial mechanism of nano-Ag materials that revealed distinct mechanisms, such as blocking biochemical pathways, DNA damage, protein complexes, and membrane disruption [4, 9].

In another instance, Xiu et al. [8] suggested that the low composition of nano-Ag or Ag ions might be causing a hermetic reaction with *E. coli* growth. Concomitantly, nano-encapsulation of NPs in bioactive forms has been a reliable and efficient method of increasing antimicrobial activity resulting in elevating the number of incorporated compounds in the liquid–solid interfaces or water-rich phases. These sites are significantly favored microbial growth, hence, specifically targeted by M-NPs [14, 15]. Furthermore, nano-encapsulation of bioactive compounds enhances the physical stability of compounds and defends them from food-pathogen interactions [14, 15].

#### ZnO-based NPs

The application of ZnO-NPs enhances efficient food packaging due to UV blocking and being cheaper than Ag-NPs shows improved antimicrobial activities. The absorption of ZnO-NPs in films can significantly increase its packaging features including durability, blockage properties, and mechanical strength [116–120]. The PLA (polylactic acid) matrix and ZnO-dispersed NPs are effectively decreased the permeability efficiency of penetrant molecules (PLA enhancing the efficiency of the diffusive route). Moreover, these eco-friendly packaging components are also demonstrated bactericidal activity. In another instance, Prakash et al. [118] demonstrated that the alteration of the film derived from bacterial cellulose through BC-PPy-ZnO (polypyrrole-Zinc oxide) nanocomposite as active and smart food packaging. It increases food storage by restricting the pathogen growth in meat products.

ZnO-NPs derived from *Catharanthus roseus* (leaf extract) performed significant antibacterial properties against *B. thuringiensis, S. aureus, P. aeruginosa, E. coli P. aeruginosa, B. subtilis,* and *C. jejuni* were much sensitive to these NPs (Table 3). ZnO-NPs showed antibacterial activity on both gram-negative and gram-positive bacteria.

Zn-NPs also exhibited antifungal activity against *P. expansum* and *Botrytis cinerea* in an elevated dose of more than 3 mmol/L. These results suggested that *P. expansum* is more sensitive than *B. cinerea* [117–119]. These NPs are a good antimicrobial agent for food packaging in micro-concentration against foodborne pathogens like *Klebsiella pneumonia, S. aureus, P. aeruginosa,* and *E. coli*. These can be described using techniques such as transmission electron microscopy (TEM) transform infrared (FT-IR) spectroscopy, ζ-potential, magnetic resonance spectroscopy, and dynamic light scattering [116].

#### Cu-based NPs

Nanocomposites of Cu polymer inhibit the growth of bacterial cells via various methods including degradation of cell wall components/carboxyl/amines in N-acetylmuramic acid and N-acetylglucosamine in the peptidoglycan layer and sulfhydryl groups on cell membranous proteins. Such destabilization in membrane proteins at cell walls subsequently degrades the bacterial membrane, cell wall components genetic structures, and generates oxidative species production causes bacterial death. Similarly, CuNPs binds to the surface of the bacterial cells via various molecular interactions and electrostatic like adherent forces. Secondly, these encapsulated NPs penetrate the cell through the membrane or outer cell surface through direct diffusion and endocytosis [120–123].

The developed Ag–Cu NPs based nanocomposite showed improved oxygen barrier efficacy, UV light, and mechanical strength. However, the film confined antimicrobial activity against *S. typhimurium* and *L. monocytogenes* were also more effective against gram-positive and gram-negative bacteria [124]. Bimetallic Ag–Cu NPs and PLA composite are prepared films along with cinnamon oil. The efficacy of the film was evaluated against *L. monocytogenes, C. jejuni,* and *S. typhimurium.* The pathogenic growth was remarkably declined with the addition of 50% cinnamon essential oil packaging [124].

Interestingly, the metal-oxide polymers are prepared using nanocomposite films, poly-ε- caprolactone biodegradable compound, disposed of ethylene terephthalate oil and zinc oxide-copper oxide have indicated efficient mechanical properties in domestic packaging. Such copper-polymer nanocomposite films have already demonstrated antimicrobial activity against *E. coli*, *Streptococcus spp., S. aureus, Pseudomonas spp.,* and *S. cerevisiae* [125].

#### Other M-NPs used in food packaging

Mixing of different metal-polymers matrices indicates various properties including structure functionalities, highly regularized pores, ordered crystalline structures, and large surface areas, provides a new insight of blending components for useful applications [123, 126]. In a published study, the Ag-doped TiO_2_ colloids showed antimicrobial activity against *C. albicans, S. typhimurium, B. subtilis, S. aureus,* and *E coli.* The fusion of Au-NPs and Ag-NPs encapsulated within chitosan films, showed promising antimicrobial properties that suppress microbial growth like *C. albicans, P. aeruginosa, S. aureus, Aspergillus niger* [127, 128]. A combination of vinyl alcohol/titanium/chitosan/ nanocomposite based on TiO_2_-NPs reduced the food spoilage microbes and helped in the storage of cheese after packaging (Table 3) [129]. Moreover, information on the application and characteristics of M-NPs are summarized in Fig. 3.

## Promises of M-NPs as antimicrobial agent

The possible antimicrobial activity of NPs may follow any of the mechanisms such as oxidizing cell components, production of secondary products such as heavy metal ions, or ROS. These directly interact with cells of microbes by penetration or disrupting the cell envelope or interfering in transmembrane electron transfer. All these alterations ultimately ended with cell death [84, 85].

Ag-NPs are the most widely used antimicrobial agent in the field of nanotechnology against multiple pathogenic strains, viruses, and fungi (Table 3) [130, 131]. Ag-NPs target the outer and cytoplasmic membranes, disrupt the function of respiratory chain enzymes, triggers the production of ROS, and can induce the bacteriostatic effect by binding Ag to the protein, enzymes, and DNA [132–134]. The noteworthy antimicrobial activity of Ag-NPs in the agar films was reported by Rhim et al. [135] against the *E. coli* and *Listeria monocytogenes*. In another similar report, a strong antimicrobial activity was documented against the *Salmonella* spp by PLA/Au-OMMT nanocomposite.

The different matrix components with M-NPs showing effectiveness against microbial growth and maintain food quality via regulating metabolic cascades are explained in Table 4 [133]. The antimicrobial effect of cellulose/chitosan nanocomposite films and cellulose/chitosan silver was examined showing the higher antimicrobial activity in films with Ag-NPs [134–137]. The Ag-NPs incorporated on films of sodium alginate were implemented on the food packing against *E. coli* and *S. aureus* [138]. The size of the Ag particle is very important for antimicrobial efficacy. The grapheme oxide is incorporated over Ag-NPs where the size is regulated by temperature and AgNO_3_ concentration. These nanohybrids showed antimicrobial properties against the *Pseudomonas aeruginosa, E. coli, Staphylococcus aureus,* and *Candida albicans* (Table 3) [139].

**Table 4:** Matrix components used during migration of metal particles and their effect on the growth of microbes

Matrix	Nanomaterial	Size	Analytical method used	Amount used	Migration amount	Effect	References
PE film PVA-Chitosan film	TiO_2_ TiO_2_	20-80 nm 17-170 nm	ICP-MS ICP-MS	250 µg/g Ti 0.1%w/v	AA: 0.23, 1.1, 2.0 E: 0.08, 0.10 and 0.35 Traces (4 × 10^–3^)	Suppress growth of *Pseudomonas, Rhodotorula,* and micro-organisms growth in packed pears UV barrier properties, reduction in the growth of for *Salmonella, E. coli,* and *S. aureus*	[187]
PLA	TiO_2_ and Ag	Nanosize	ICP-AES	0.5% w/w	AA < 0.59 E < 0.17	Inhibits physiological processes (respiration, ethylene production), growth of yeast, molds and maintain the quality of bayberries	[189]
PLA	TiO_2_ and Ag	10-15 nm	ICP-AES	3w/w	H:18.8–21.2	Hinder the oxidation of fish oil	[190]
LDPE	ZnO	20-400 nm	–	0.25% w/w	< 0.12	Inhibit the growth of *Salmonella* in liquid egg	[191]
LDPE	ZnO and Ag	70 nm ZnO, 10 nm Ag	ICP-MN	10%w/w	Zn-407, Ag < 0.17	Reduce the growth of *E. coli* and prevent phytodegradation	[192]
LDPE	CuO	50 nm	AAS	1%w/w	38	Decrease the growth of coliform bacteria and reduce microbial growth	[193]
PLA	Al_2_O_3_	25 nm	ICP-MS	25 nm coating	Al-500	Improve gas barrier properties	[194]
LDPE	Ag, TiO_2_ and SiO_2_	40–60 nm	ICP-MN	1% w/w	–	Lowers the respiration rate, ethylene scavenging and maintain the nutrient quality	[195]

The CuNPs showed antimicrobial activity after the 4-h exposures against the *Saccharomyces cerevisiae, S. aureus, E. coli,* and *L. monocytogenes* [140]. The polyurethane nanofibers containing CuNPs was showing higher antibacterial activity against the *B. subtilis* and *E. coli* [141]. CuNPs are having several toxic effects including ROS production, protein oxidation, lipid peroxidation, and DNA degradation [142]. The ZnNPs on incorporation with the plastic matrix have antifungal and antimicrobial activity [143].

Other antimicrobial NPs such as MgO, TiO_2_, and ZnO are also having a role in food packaging by showing photo-catalytic disinfection and UV blockers. TiO_2_-NPs are the most promising one among all. These are active under the UV light radiations and display antimicrobial activity against *Vibrio parahaemolyticus*, *S. choleraesuis*, and *L. monocytogenes* only in light conditions [144–148]. The probable mechanism of action of TiO_2_ as the antibacterial activity involves the attack of oxidative entities on the inner or outer cell membrane of bacteria, DNA damage, and also the altered *Coenzyme A* dependent activity [149].

The photoactivated biocidal activity TiO_2_ was reported against the nine bacterial species such as *Bacillus* sp., *B. stearothermophilus*, *Erwinia caratovora*, *E. coli*, *L. plantarum*, *P. fluorescens*, *P. jadinii*, *S. aureus*, and *Z. rouxii* [147]. The TiO_2_-coated plastic films showed antimicrobial activity against the *Penicillium expansum* on the spoilage of lemons, apples, and tomatoes [151]. Furthermore, ZnO has also shown better antibacterial activity with the reduction in particle size and also required visible light for activation [149, 150]. It induces the destruction of cell wall integrity, shoots in ROS production, and liberation of Zn^2+^ antimicrobial ions [151]. Similarly, ZnO was observed to be the most effective antibacterial agent against *S. aureus* (Fig. 2).

## Health risks, toxicity, and drawbacks of using NPs for food packaging

**Fig. 4:**
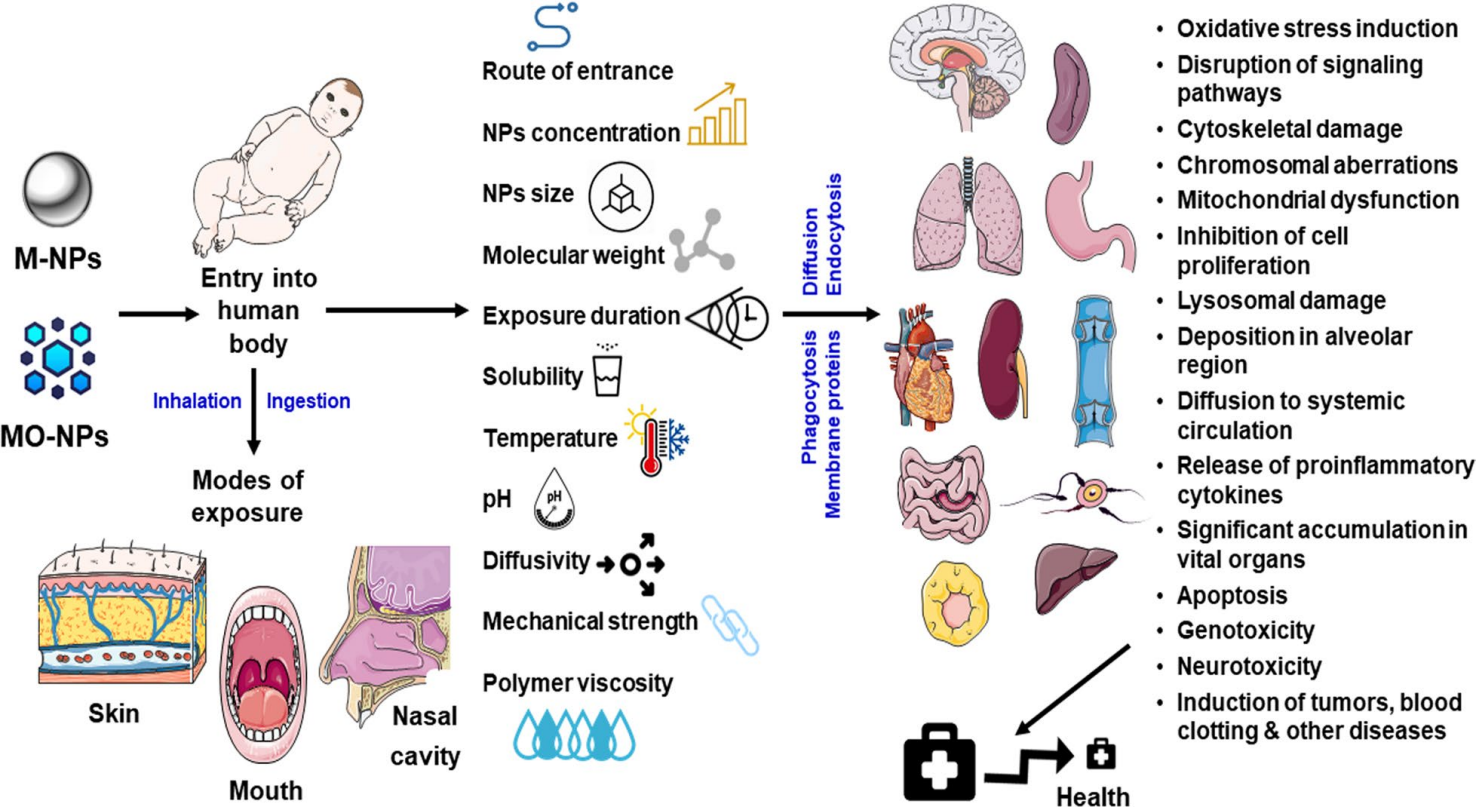
Negative impacts of M-NPs based packed food consumption on human health

Despite a hundreds of papers published in nanotechnology to date, still, numerous publications focus more on the negative impacts of NPs. As per the literature, ingestion, inhalation, or accidental exposure of M-NPs during packaging and processing creates immense health risks in workers [152–154]. It even includes lysosomal damage, cardiovascular diseases, lung tumors, blood clotting, and induced cytotoxicity (Fig. 4) [152–154].

In this context, the spleen and liver are chiefly responsible for the passage of NPs, guiding their transportation from the intestine to the bloodstream. Studies on Ag- and TiO_2_-NPs demonstrated that these substances are insoluble and significantly accumulated in organs [155]. Such unintentional migration through packaged products may raise several serious health concerns to even consumers. The migration of these M-NPs solely depends upon the chemical and physical properties of polymer complexes and food [156]. Additionally, several other regulating parameters such as particle size, solubility, molecular weight, concentration, polymer viscosity, temperature, pH value, the diffusivity of a compound in polymer, contact time, mechanical strength, composition, and matrix of food. However, concentration, route of an entrance, toxicity, and duration of exposure also affect the state and susceptibility of an organism [157].

In comparison, NPs increase their possibilities of interaction that ultimately lead to various hazardous effects. For instance, nano-TiO_2_ and nano-Ag induced high toxicity causes carcinogenicity and genotoxicity, even larger-sized ZnO is less toxic indicating the role of NPs diameter. Inhalation of Nano-TiO_2_ (10 mg/m^3^) enhances the risk of lung tumor. ZnO at 10 μg/ml concentration inhibits the cell proliferation, viability of Caco-2 cells concentration, and elevates ROS production along with super oxidase levels; implies an oxidative stress response (Fig. 2) [158]. Few studies had reported that enhanced TiO_2_-NPs can distort the structure of microvilli in epithelial cells of intestinal tissue and affects the normal nutrient absorption. Moreover, systemic inhalation of MgO-NPs migrates to the olfactory bundle via sensory nerves cause an ill effect on various parts of the brain. These NPs become migratory via the bloodstream and may affect the lining of blood vessels even crossing blood–brain barriers in the fetus [159].

In this context, the regulatory system is crucial to access the significant risks that emerge by the use of MNPs in the food sector [2, 158]. According to European Commission regulation (ECR), number 2015/2285, the European food safety authority (EFSA) must confirm that for the association of engineered NPs in food safety assessment was carried out using the modern generation of analytic approaches. In the united states (US), 258/97 regulation stipulates that if NPs used as active ingredients it has to be definitely used for fresh food. Another regulation no. 133/2008 by European commission term “food additive and its composition” as evaluation mark, as a new and safe material before used commercially [2, 3].

Besides these issues, M-NPs usage triggered non-biodegradable concerns affecting environmental health creates eco-toxicity leading to a vague situation for all exciting communities on the planet. Nevertheless, additional meticulous information and scientific data are required at this infancy stage of M-NPs applications in food packaging [202–204]. Although, toxicity during migration, distribution, absorption, metabolism and finally their everlasting residues should be evaluated and quantified the risk assessment to the consumer level [85].

## Perceptions and future perspectives

Since its inception, nanotechnology shows extensive and fruitful results in developing new products but enhances safety issues that deserve well attention. Therefore, understanding numerous techniques with diversified roles draws many variegate and safety conclusions regarding toxicity. The assessment of recent development in M-NPs plays a substantial function and the assessment of adverse outcomes. Several benefits of projected food nanotechnology like the use of stable emulsions, less fat, better taste in mayonnaise, ice-cream, canned stuff taste masking of additives including fish oils, enhanced level of nutrients, optic appearance, antimicrobial action, supplements, and other nano-textured food prepared after killing of pathogenic microbes. It also offers flexibility in packaging, moisture, temperature, gas barrier activities and helps in maintaining active packaging using M-NPs. Furthermore, it inhibits the growth of microbial pathogens on the plastic surface. No doubt, these second-generation NPs add flexibility in preservation activity, antimicrobial activities, binding bioavailability with desired food and polymer extract without hinders to the mechanical strength, gasses movements, and flavors.

Moreover, water filtration, food clarification, plant extract waste, and other undesired components still need more progression in food industry research. The degradation, transformation of carbon-based carrier substrates during metal incorporation in food products and the environment remains a prime challenge toward acceptance of these techniques. Recently, fresh fruit cutting, food packaging, processing, storage, transportation-related industries have rapidly enhanced. These M-NPs based applications help in the improvement of food packaging to sustain food preservation and shelf life (Table 5).

**Table 5:** Role of Metal complexes and their successful application in food packaging

M-NPs/Polymeric complex	Targeted microbes	Successful food application	References
Cellulose-Ag-NPs HPMC-Ag-NPs PVC-Ag-NPs EVOH -Ag-NPs	Aerobic bacteria, lactic acid bacteria, and *Pseudomonas* sp. *E. coli, S. aureus* *S. aureus, E. coli,* and total mesophilic bacteria *L. monocytogenes* and *Salmonella* sp*.*	Beef-meat – Beef Cheese, chicken	[196]
Cellulose-Ag-NPs Calcium alginate/Ag-montmorillonite NPs	Aerobic bacteria, Psychotropic bacteria, Yeasts, and molds Aerobic bacteria	Freshly-cut melon Freshly-cut carrot	[181]
Pullulan-Ag-NPs	*L. monocytogenes* and *S. aureus* *L. monocytogenes* and *S. aureus*	Turkey meat Poultry products, meat	[42]
Pullulan-ZnO-NPs	*E. coli*, *L. monocytogenes,* and *S. aureus*	Poultry products, meat	[197]
Agar hydrogel, Ag-montmorillonite NPs Sodium alginate-CaCl_2_/ Ag-NPs LDPE-Ag-NPs/ZnO-NPs Cellulose-Cu NPs Oriented PP. coated film, TiO_2_ Polyurethane Ag-NPs	*Pseudomonas* *–* *Pseudomonas, Enterobacter* and *Escherichia coli* Yeast, mold, and total aerobic bacteria *S. cerevisiae, S. epidermis* and *S. aureus* *S. aureus* and *E. coli* *S. aureus* and *E. coli*	Cheese – Cheese Orange juice Fruit juice Lettuce Lettuce	[198]
Sodium alginate-Ag-NPs Hydroxypropyl methylcellulose Cu NPs Chitosan-TiO_2_	*E. coli* and *S. aureus* *S. aureus, B. aureus* and *Salmonella* sp*.* *S. aureus, Salmonella sp.* and *E. coli*	Pears Meat Cheese, chicken	[199]
LDPE/Ag-NPs LDPE-Ag-NPs/TiO_2_ Polyethylene-Ag/ TiO_2_-NPs Polyethylene -Ag/TiO_2-_NPs HDPE/Marigold extract-TiO_2_	Aerobic bacteria Aerobic bacteria Lac*tobacillus* and *Penicillium* *S. aureus* and *E. coli* *Salmonella* sp*.*	Barberry Strawberry Fresh-apple, carrots, milk powder White slice bread, cheese, milk powder Soybean oil	[153]
Whey protein, cast film, TiO_2_	*S. aureus* and *Salmonella* sp*.*	Meat	[200]
LDPE /Blown film TiO_2_	*Pseudomonas, Rhodotorula*	Almonds	[201]

The advancement of M-NPs requires more research that is not limited to packaging and processing but expands toward quality traits, nutraceutical values, and natural flavors of the product. The transfer of metal particles along with the foodstuff is a primary step. It needs more intense clarification and validation on the disadvantages such as persistence of metal ion, cost-effectiveness, uncontrolled bioactive release, toxicity percentage, stability inactivity after migration, and public acceptance.

## Concluding remarks

With the advent of awareness of health, nutrition, food safety, and global food demand, a huge demand for adopting new technologies to enhance the quantity and quality of food products has emerged. The importance develops from the M-NPs (ZnO, SiO_2_, CeO_2_, TiO_2_, Ag-NPs, and Au) nanoscale characteristics, oxygen scavengers, physicochemical properties, and antimicrobial activities. M-NPs are efficient antimicrobial agents and effectively inhibit the growth of a diverse range of food-borne pathogenic bacteria, mold, and fungi. Polymers used for encapsulation of M-NPs play a significant role as a carrier vehicle without hindering the natural properties. These polymer-metal complexes are assumed to be promising in increasing shelf life and are ecologically essential for food packaging. Additionally, such post-processing technologies at the nanoscale level offer direct incorporation of antimicrobial compounds in space around the foodstuffs and ensure their quality during storage at the long interval.

Furthermore, active packaging using biocidal substances overcomes the challenges of target-based delivery, the life span of the product, nutrients degradation, and stability of packaging compounds. Intensive research has evaluated the significant role of improving food quality, toxicity monitoring, and degradation after transport to help in understanding functional properties. Despite these, the intake of metallic particles with food creates potential health issues and is always at risk of public rejection. Moreover, toxicological aspects and resistance of microbes toward M-NPs still need extended research validations. The nanotechnologies and their potential uses need a standard regulatory framework and labelling of food to address the consumer demands and their commercial adoption to reduce nano-enabled food. In summary, toxicity concerns have raised numerous questions about potential implementation to ensure the safe use of M-NPs in food industries.

## Data Availability

Not applicable.

## Abbreviations

NPs: Nanoparticles
M-NPs: Metal-based nanoparticles
FAOs: Food and agriculture organization
FLI: Food Loss Index
UNEP: United nations environment programme
FWI: Food waste index
WHO: World health organization
CNTs: Carbon nanotubes
PET: Polyethylene terephthalate
PP: Polypropylene
PE: Polyethylene
AgNPs: Silver nanoparticles
Silica NPs: Si NPs
nano-ZnO: Nano Zinc oxide
nano-Ce_2_O_4_: Nano cerium oxide
nano-TiO_2_: Nano titanium oxide
nano-Ag: Nano silver: nanosilver
Au: Gold
Ag: Silver
Zn: Zinc
TiO_2_: Titanium oxide
SiO_2_: Silicon oxide
MgO: Magnesium oxide
ZnO: Zinc oxide
PCR: Polymerase chain reaction
AgNO_3_: Silver nitrate
NaBH_3_: Sodium cyanoborohydride
ZnO*–*SnO_2_: Zinc oxide Tin oxide
BSA: Bisphenol A
MOs: Metal oxides
MMOs: Mixed metal oxides
UV: Ultraviolet
PLA: Polylactic acid
CuO: Copper oxide
Pd: Palladium
Pt: Platinum
Ag: Silver
Au: Gold
Ti: Titanium
N: Nitrogen
Mg-ONPs: Magnesium oxide nanoparticles
CNTs: Carbon nanotubes
Au-NPs: Gold nanoparticles
Cu-NPs: Copper nanoparticles
CuO-NPs: Copper oxide nanoparticles
MgO-NPs: Magnesium oxide nanoparticles
IHPC: Institute for Health and Consumer Protection
ROS: Reactive oxygen species
POD: Pyrogallol peroxidase
MDA: Malondialdehyde
OMMT: Organic montmorillonite
Zn^2^^+^: Zinc
BC-PPy: Bacterial cellulosepolypyrrole
Cu: Copper
Zn: Zinc
Ag: Silver
PE film: Polyethylene film
ICP-MS: Inductively coupled plasma mass spectrometry
PVA: Polyvinyl allyl
PLA: Poly-lactic acid
ICP-AES: Inductively coupled plasma atomic emission spectroscopy
LDPE: Lowdensity polyethylene
CaCl_2_: Calcium dichloride
PVC: Polyvinyl chloride
EVOH: Ethylene vinyl alcohol
HDPE: High Density Poly Ethylene
GC–MS: Gas chromatography-mass spectroscopy
Au@AgNP: Gold coated silver nanoparticles
MWCNT: Multiwalled carbon sanotubes
SERS: Surface enhanced Raman spectroscopy
ECR: European commission regulation
US: United states
EFSA: European food safety authority
